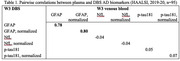# Assessing the feasibility of dried blood spot (DBS) collection for blood‐based biomarkers of Alzheimer’s Disease in rural South Africa

**DOI:** 10.1002/alz.092596

**Published:** 2025-01-09

**Authors:** Sarah Gao, Darina T. Bassil, Ryan G. Wagner, Jacques du Toit, Hanna Huber, Nicholas J. Ashton, Dejania Cotton‐Samuel, Meagan T. Farrell, Victor Mngomezulu, Jennifer J. Manly, Stephen Tollman, Lisa F. Berkman, Henrik Zetterberg, Adam M. Brickman

**Affiliations:** ^1^ Harvard T. H. Chan School of Public Health, Cambridge, MA USA; ^2^ Harvard T.H. Chan School of Public Health, Cambridge, MA USA; ^3^ University of the Witwatersrand, Johannesburg South Africa; ^4^ University of Gothenburg, Mölndal Sweden; ^5^ Department of Psychiatry and Neurochemistry, Institute of Neuroscience and Physiology, The Sahlgrenska Academy, University of Gothenburg, Mölndal, Gothenburg Sweden; ^6^ Taub Institute for Research on Alzheimer's Disease and the Aging Brain, New York, NY USA; ^7^ G.H. Sergievsky Center, Vagelos College of Physicians and Surgeons, Columbia University, New York, NY USA; ^8^ Department of Neurology, Vagelos College of Physicians and Surgeons, Columbia University, New York Presbyterian Hospital, New York, NY USA; ^9^ IQVIA, New York, NY USA; ^10^ Department of Neurology, Vagelos College of Physicians and Surgeons, Columbia University, and the New York Presbyterian Hospital, New York, NY USA; ^11^ Department of Neurology, Columbia University Irving Medical Center, New York, NY USA; ^12^ Taub Institute for Research on Alzheimer’s Disease and the Aging Brain, Columbia University, New York, NY USA; ^13^ The Gertrude H. Sergievsky Center, College of Physicians and Surgeons, Columbia University, New York, NY USA; ^14^ Department of Neurology, Columbia University, New York, NY USA; ^15^ Department of Neurology, College of Physicians and Surgeons, Columbia University, New York, NY USA; ^16^ Columbia University, New York, NY USA; ^17^ Institute of Neuroscience and Physiology, Sahlgrenska Academy at the University of Gothenburg, Mölndal, Gothenburg Sweden; ^18^ UCL Institute of Neurology, Queen Square, London United Kingdom; ^19^ Institute of Neuroscience and Physiology, The Sahlgrenska Academy at the University of Gothenburg, Mölndal Sweden; ^20^ Institute of Neuroscience and Physiology, Sahlgrenska Academy at the University of Gothenburg, Gothenburg Sweden; ^21^ Hong Kong Center for Neurodegenerative Diseases, Hong Kong China; ^22^ Theme Aging, Karolinska University Hospital, Stockohlm Sweden; ^23^ University of Gothenburg, Mölndal, Gothenburg Sweden; ^24^ Sahlgrenska University Hospital, Gothenburg Sweden; ^25^ University of Gothenburg, Gothenburg Sweden; ^26^ Dementia Research Centre, Department of Neurodegenerative Disease, UCL Queen Square Institute of Neurology, University College London, London, United Kingdom, London United Kingdom; ^27^ UK Dementia Research Institute at UCL, London United Kingdom

## Abstract

**Background:**

The characterization of Alzheimer’s disease (AD) and AD related dementias (ADRD) pathophysiology has been revolutionized by the development of highly sensitive blood‐based biomarkers. Although blood‐based biomarkers allow for greater access, cost effectiveness, and scalability, there are limitations for their implementation in resource‐constrained low‐ and middle‐income countries (LMICs) and rural settings, where access to equipment, freezers, and assays is often limited. Dried blood spot (DBS) collection emerges as a promising, convenient, and cost‐effective method for acquiring blood samples in these contexts, but it is unclear whether highly sensitive assays typically applied to cerebrospinal fluid (CSF), plasma, or serum can detect biomarker concentrations accurately. This pilot study assessed the feasibility of using DBS to measure blood‐based AD biomarkers by examining the agreement between ADRD biomarker concentrations derived from DBS and from plasma in older adults in rural South Africa.

**Method:**

Data were collected from the Health and Aging in Africa: A Longitudinal Study of an INDEPTH Community in South Africa (HAALSI), an ongoing population cohort of 5,059 adults aged ≥40 years living in Agincourt, South Africa. This study focuses on a sub‐sample of 95 participants, whose venous blood and DBS were collected during the HAALSI visit and later tested for blood‐based ADRD biomarkers, including neurofilament light chain (NfL), phosphorylated‐tau181 (p‐tau181), and glial fibrillary acidic protein (GFAP). Plasma and DBS markers were analyzed using commercially available Single molecule array assays (Quanterix) at the University of Gothenburg. We ran pairwise correlations that examined the association between the plasma and DBS measures of p‐tau181, NfL, and GFAP.

**Result:**

The sample consisted of 70.5% women, with an average age of 67.53 years (SD=9.69). We observed a positive correlation between plasma and DBS GFAP concentrations (r=0.78, p‐value<0.001). However, DBS and plasma concentrations were not correlated for p‐tau181 (r=0.05, p‐value=0.62) or for NfL (r=‐0.04, p‐value=0.68).

**Conclusion:**

These preliminary analyses suggest that GFAP concentration, a marker of astrogliosis, can be measured consistently in DBS. These findings have important implications for the selection and analysis of AD blood‐based biomarkers in low‐resource and LMIC settings, where venous blood draws may not be feasible.